# From fixed time points to personalized care: rethinking stroke risk management in atrial fibrillation patients

**DOI:** 10.1093/europace/euaf215

**Published:** 2025-09-11

**Authors:** Davide Antonio Mei, Jacopo Francesco Imberti, Giuseppe Boriani

**Affiliations:** Cardiology Division, Department of Biomedical, Metabolic and Neural Sciences, University of Modena and Reggio Emilia, Policlinico di Modena, Via del Pozzo 71, Modena 41121, Italy; Clinical and Experimental Medicine PhD Program, University of Modena and Reggio Emilia, Modena, Italy; Cardiology Division, Department of Biomedical, Metabolic and Neural Sciences, University of Modena and Reggio Emilia, Policlinico di Modena, Via del Pozzo 71, Modena 41121, Italy; Cardiology Division, Department of Biomedical, Metabolic and Neural Sciences, University of Modena and Reggio Emilia, Policlinico di Modena, Via del Pozzo 71, Modena 41121, Italy

**This editorial refers to ‘Timing of stroke risk reassessment in atrial fibrillation patients with CHA_2_DS_2_-VA score of 0 or 1: the Norwegian AFNOR study’ by M. Anjum *et al*., https://doi.org/10.1093/europace/euaf145.**

Since the earliest clinical observations, atrial fibrillation (AF) has been strongly associated with an increased risk of stroke.^1^ This robust association established oral anticoagulation (OAC) as the cornerstone of stroke prevention in AF management. Consequently, the accurate definition of each patient’s thromboembolic risk profile, together with bleeding risk, has become fundamental for informed decision-making. This need led to the widespread adoption of risk scores as tools to facilitate clinical decision-making, both for thromboembolic and bleeding risk stratification.^1^

The most recent European guidelines recommend the CHA₂DS₂-VA score for thromboembolic risk assessment.^2,3^ This represents an important change from the previous version of the guidelines, which endorsed the CHA₂DS₂-VASc score, mainly characterized by removal of female sex as an independent risk factor for stroke.^4^ This shift prompted several subsequent studies, which consistently showed that in recent years the two scores became essentially comparable in terms of predictive performance, both for the risk of incident stroke and for residual stroke risk,^5,6^ with the advantage of a simpler decision-making by adoption of CHA₂DS₂-VA.^3,4,6^

Regardless of which score is adopted, the fundamental role of scores has been, and continues to be, the identification of patients at truly *low risk of stroke*, for whom anticoagulation is not needed or may be object of careful consideration.^1^ In clinical practice, the CHA₂DS₂-VASc score (and now CHA₂DS₂-VA) has been particularly valuable in ruling out patients who can safely remain untreated, avoiding both overtreatment and bleeding complications.

In these terms, guidelines are clear when it comes to the initial management of stroke prevention for AF individuals; they advise to assess the individual stroke risk, and when this risk is sufficiently elevated (CHA₂DS₂-VA ≥ 2, class I, level of evidence A), initiate anticoagulation therapy. Alongside this, clinicians are advised to weigh bleeding risk not with the aim to withhold or stop anticoagulation, but rather to identify and address modifiable factors. This is particularly important given the known limitations of current bleeding risk scores in accurately predicting clinical events.^7^

After this first evaluation, most guidelines suggest periodic reassessment—typically on an annual basis, though earlier follow-up may be considered at the physician’s discretion.^2^ What remains much less clear, however, is how to manage patients who are initially categorized as low risk (CHA₂DS₂-VA = 0) or those at intermediate risk (CHA₂DS₂-VA = 1) in whom the decision may be not to initiate anticoagulation, according to lack of a class I recommendation (for CHA₂DS₂-VA = 1, the indication to OAC has a class IIa recommendation, level of evidence A, leading to individual assessment).^8^ Very few guidelines provide explicit recommendations for these individuals (*Table 1*). However, these patient groups deserve special attention. Both AF and the clinical profile are dynamic conditions, and patients inevitably may accumulate additional thromboembolic risk factors over time. For those who start with a low baseline risk, timely recognition of this progression is crucial. Leaving patients unprotected by OAC therapy while their risk silently increases exposes them to strokes and potentially devastating outcomes that could be prevented by OAC (*Figure 1*).

**Figure 1:**
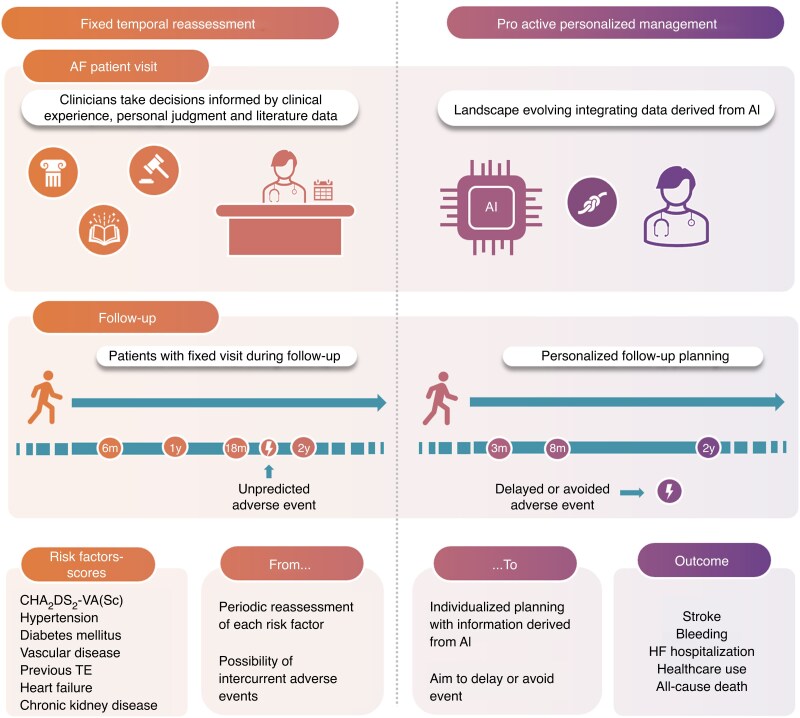
Potential shift in AF management from fixed time points for clinical profile reassessment to personalized follow-up. AF, atrial fibrillation; AI, artificial intelligence; HF, heart failure; m, months; TE, thromboembolic events; y, years.

**Table 1:** Indications to reassessment of stroke risk (with corresponding level of evidence and recommendation) according to latest version of AF guidelines of different associations

	CHA_2_DS_2_-VA ≥ 2	CHA_2_DS_2_-VA 0–1
2018 National Heart Foundation of Australia and the Cardiac Society of Australia and New Zealand	NR	Annually (suggested)
2020 Canadian Cardiovascular Society and Canadian Heart Rhythm Society	Annually (strong recommendation moderate-quality evidence)	NR
2021 National Institute for Health and Care Excellence (NICE)	Annually (suggested)	NR
2021 Asia Pacific Heart Rhythm Society	Annually (or every 4 months) (recommended)	4 months (should be done)
2023 American College of Cardiology, American Heart Association, American College of Chest Physicians and Heart Rhythm Society	Annually (Class 1, LoE B)	NR
2024 Chinese Society of Cardiology, Chinese Medical Association, Heart Rhythm Committee of Chinese Society of Biomedical Engineering	Annually (Class 1, LoE C)	Annually (Class 1, LoE C)
2024 European Society of Cardiology and European Association for Cardio-Thoracic Surgery	Annually (suggested)	NR

LoE, level of evidence; NR, not reported.

Modern clinical guidelines attempt to address this challenge by recommending fixed-time reassessments, but only a minority of guidelines worldwide provide detailed indications on the timeline of clinical re-evaluation (*Table 1*). Moreover, reassessment is almost universally based on fixed time points: the clinician determines when to review the patient, and risk is reassessed at that interval. This approach, while pragmatic, may not fully capture the dynamic nature of AF and its associated comorbidities. Data on the *optimal timing* of reassessment, particularly in European populations, have been lacking.

In this context, the study by Anjum *et al*.^9^ published in the current issue of *EP Europace*, represents a timely and clinically relevant contribution. Using Norwegian national registries from 2011 to 2018, the authors identified 40,782 patients with incident AF and a low (CHA₂DS₂-VA = 0) or intermediate (CHA₂DS₂-VA = 1) risk profile. Patients were followed from the time of AF diagnosis until their CHA₂DS₂-VA score increased. The investigators then calculated the proportion of patients whose risk progressed over time and estimated the number needed to reassess (NNR) to detect one new risk factor at different time intervals.

Half of patients experienced an increase in their CHA₂DS₂-VA score within a median follow-up of only 1.7 years. Risk progression occurred in 19% of patients at 6 months, 25% at 1 year, and 40% at 3 years. Age implied a variable association with progression of the risk profile for stroke: at 1 year, only 8% of patients aged 18–44 years and 14% of those aged 45–54 years had acquired a new risk factor, compared with 30% in patients older than 55 years. This translated into an NNR at 1 year of 12, 7, and 3, respectively. Based on these results, the authors propose age-tailored reassessment: every 6 months for patients ≥ 55 years, annually for those <55 years, with fixed visits at the transition ages of 65 and 75 years.

These results are important and provide practical, data-driven guidance for tailoring reassessment intervals to patients’ baseline risk, thereby refining current practice. Informing clinicians about the optimal timing to detect the emergence of new comorbidities is indeed essential in the management of AF patients. Notably, a prior study^10^ showed that longitudinal changes in CHA₂DS₂-VASc (the so-called delta score) were stronger predictors of ischaemic stroke than either baseline or follow-up values alone. This finding underscores the prognostic significance of risk evolution over time and highlights the clinical value of the present study. However, while this work represents an important step forward towards a more personalized management of AF patients, it still relies on the use of fixed reassessment intervals. Furthermore, as acknowledged by the authors themselves, the study did not evaluate the impact of such a strategy on clinical outcomes or on the rates of OAC prescription. In this perspective, the near future may require a true paradigm shift towards a more personalized model of follow-up (*Figure 1*). The priority should be to refine patient trajectories as much as possible, with the primary goal of anticipating, or even preventing, events, rather than simply reacting to them.

Emerging strategies, such as prediction models based on artificial intelligence (AI), may help to detect early, subtle changes in a patient’s risk profile and predict the trajectories of future evolution.^11^ Such dynamic monitoring might allow clinicians to intervene precisely when the balance tips in favour of anticoagulation, instead of waiting for an arbitrary interval to elapse. Dedicated initiatives, such as the ARISTOTELES project^12^ funded by the Horizon programme, are already exploring whether AI can enhance the management of AF patients by predicting and guiding interventions for newly developed or worsening comorbidities (*Figure 1*).

Furthermore, it is essential to stress that decisions regarding anticoagulation represent only the first (albeit crucial) step in the management of AF. Current recommendations emphasize that patients should be treated within a holistic and integrated care framework, which not only addresses stroke prevention but also ensures optimal symptom control (ranging from antiarrhythmic therapy to ablation) and comprehensive management of comorbidities.^4,13–15^

Ultimately, what is and will increasingly remain a crucial step in the care of AF patients is the ability to address the full spectrum of patient needs—across both low- and high-risk profiles. A “one-size-fits-all” approach is no longer sufficient. In this regard, the findings of the present study represent a valuable step forward towards a more precise and truly personalized management of AF patients.

## Data Availability

No data used in this study.
